# Shaping a data-driven era in dementia care pathway through computational neurology approaches

**DOI:** 10.1186/s12916-020-01841-1

**Published:** 2020-12-16

**Authors:** KongFatt Wong-Lin, Paula L. McClean, Niamh McCombe, Daman Kaur, Jose M. Sanchez-Bornot, Paddy Gillespie, Stephen Todd, David P. Finn, Alok Joshi, Joseph Kane, Bernadette McGuinness

**Affiliations:** 1 Intelligent Systems Research Centre, School of Computing, Engineering and Intelligent Systems, Ulster University, Magee Campus, Londonderry, Northern Ireland, UK; 2 Northern Ireland Centre for Stratified Medicine, Biomedical Sciences Research Institute, Ulster University, Magee Campus, Londonderry, Northern Ireland, UK; 3 Health Economics and Policy Analysis Centre, Discipline of Economics, National University of Ireland, Galway, Ireland; 4 Altnagelvin Area Hospital, Western Health and Social Care Trust, Londonderry, Northern Ireland, UK; 5 Pharmacology and Therapeutics, School of Medicine, Galway Neuroscience Centre, National University of Ireland, Galway, Ireland; 6 School of Medicine, Dentistry and Biomedical Sciences, Institute for Health Sciences, Centre for Public Health, Queen’s University Belfast, Belfast, Northern Ireland, UK

**Keywords:** Dementia, Alzheimer’s disease, Dementia care pathway, Data science, Computational neurology, Computational modelling, Computational neuroscience, Healthcare economics, Clinical decision support systems

## Abstract

**Background:**

Dementia is caused by a variety of neurodegenerative diseases and is associated with a decline in memory and other cognitive abilities, while inflicting an enormous socioeconomic burden. The complexity of dementia and its associated comorbidities presents immense challenges for dementia research and care, particularly in clinical decision-making.

**Main body:**

Despite the lack of disease-modifying therapies, there is an increasing and urgent need to make timely and accurate clinical decisions in dementia diagnosis and prognosis to allow appropriate care and treatment. However, the dementia care pathway is currently suboptimal. We propose that through computational approaches, understanding of dementia aetiology could be improved, and dementia assessments could be more standardised, objective and efficient. In particular, we suggest that these will involve appropriate data infrastructure, the use of data-driven computational neurology approaches and the development of practical clinical decision support systems. We also discuss the technical, structural, economic, political and policy-making challenges that accompany such implementations.

**Conclusion:**

The data-driven era for dementia research has arrived with the potential to transform the healthcare system, creating a more efficient, transparent and personalised service for dementia.

## Background

Dementia refers to a clinical syndrome distinct from physiological ageing, caused by one or more pathological processes and characterised by progressive impairment in cognition and everyday functioning [1]. Alzheimer’s disease (AD), typically characterised by impairment in memory, is the most common subtype of dementia, constituting 60–70% of the cases [1]. AD can be categorised as familial AD (with a family history of the disease and early AD onset) and sporadic AD, with the latter overwhelmingly being the most common type [2]. AD may co-exist with pathological processes characteristic of other common dementia subtypes such as vascular dementia, frontotemporal dementia and Lewy body dementia [1]. Further, there may also be co-morbidities with other illnesses such as epilepsy [3]. To add to the complexity, the prodromal stages, or mild cognitive impairment (MCI), associated with some dementia subtypes, can be loosely defined and heterogenous, particularly when assessments are subject to factors like delirium, psychiatric illness and the effects of medication [1, 4].

Globally, it is estimated that there were 47 million people with dementia in 2015, and with a rapidly growing ageing population, this is expected to reach 75 million by 2030 and 132 million by 2050 [5]. Dementia has a considerable impact on the well-being and functioning of those living with the disease, and also on their families and caregivers. Dementia care can place health and social care services under an operational and financial strain, costing an estimated US$ 818 billion in 2015 and estimated US$2 trillion in 2030 [5]. In the UK, dementia costs £26 billion per year. In 2014, 850,000 people in the UK were estimated to be living with dementia, and this may rise to 1.6 million by 2040 [6]. In neighbouring Ireland, there were about 48,000 people with dementia in 2011, and this is projected to increase to 132,000 by 2041, while costing €1.7 billion annually [7, 8].

Despite the demand for dementia care and treatment, to date, there are no disease-modifying therapies for the most common dementia subtypes. Medications that target particular neurotransmitter systems (e.g. cholinesterase inhibitors) and nutritional supplements have been proposed to slow the early cognitive decline associated with mild to moderate AD and Lewy body dementia [9, 10]. Trials investigating disease-modifying therapies have mostly targeted the formation of beta-amyloid plaques, suggested to be one of the neuropathological hallmarks of AD, but the results have so far been underwhelming [11, 12]. This may be attributed to testing people with dementia too late; by the time that the clinical symptoms have manifested themselves, amyloid may have been accumulating in the brain structures for several years [13, 14]. Therapies targeting hyperphosphorylated tau (twisted fibres of tau proteins), the other main neuropathological substrate of AD, have also failed to demonstrate significant improvements in clinical outcomes [13, 14]. In all likelihood, AD and other dementia subtypes are likely to be the product of interactions between multiple factors, including but not limited to cholinergic neuronal damage, neuroinflammation, oxidative stress, glucose hypometabolism and, more recently, gut microbiome perturbations via the immune system, endocrine system, vagus nerve and bacteria-derived metabolites [14]. It is also possible that some of these hypotheses could be related [15], but further confirmatory work is required.

Regardless of our incomplete understanding of dementia, the rising global population and longer average lifespan [1, 16] make an increasing and urgent case for timely and accurate recognition of dementia and its subtypes, particularly in guiding clinical decision regarding appropriate clinical care. Indeed, it is projected that the direct healthcare costs of early diagnosis may be offset by the cost savings arising from the earlier targeting of patients to the appropriate clinical care pathways [17]. Such savings may be linked to the benefits of earlier delivery of dementia medication and caregiver interventions, and delaying institutionalisation, thereby reducing the overall direct and indirect health and social care cost burden [17]. In addition, early diagnosis and intervention increases the quality of life and care planning for people with dementia and their caregivers, which promote independence [17]. In this context, it is clear that the potential economic and humane benefits of improving the clinical care pathway for dementia are immense. Indeed, as we shall discuss below, the application of data-driven computational approaches can have an immediate impact on improving dementia care pathway.

## Dementia care pathway

**Fig. 1:**
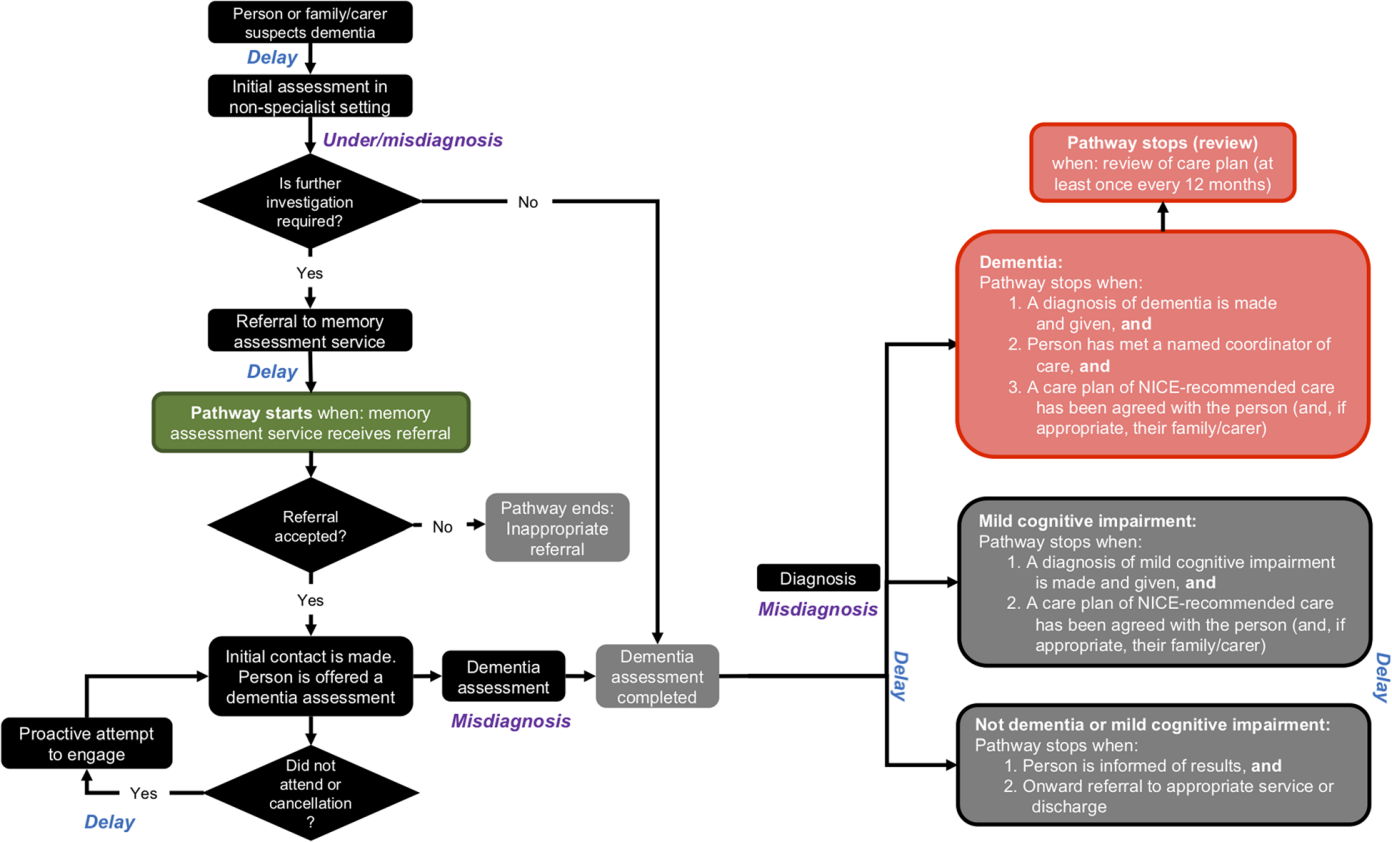
Flowchart of the UK dementia care pathway under the NICE guidelines and potential disruption. Includes primary and secondary (specialist) care. Blue and purple texts: potential time delays and under/misdiagnoses and also opportunities for technologies and novel dementia markers. Flowchart based on [19]

To evaluate the effectiveness of dementia care, we must first assess the current dementia care pathway. As an example, the pre-eminent body in the UK working on clinical guidelines and standardised practices for medical professionals is the National Institute for Health and Care Excellence (NICE), with dementia care guidelines updated in 2018 to reflect the current best practices [18]. The guidelines put forth several strong recommendations for how dementia care should be implemented at the primary care level, at specialist memory assessment services and in the wider community. A schematic of the NICE 2018 recommendations for the dementia care pathway is illustrated in Fig. 1 [19]. Symptoms of dementia are usually first identified by either the individual themselves or a family member of caregiver, before being assessed by general practitioners (GPs). At the primary care level, a major focus is to exclude common and treatable causes of delirium or other disorders. If dementia remains a concern, further investigation and onward referral to secondary care are required, where more detailed assessment by a specialist (e.g. memory clinic) will diagnose dementia, its subtype and initiate treatment [19, 20].

Two major issues that often impede the effectiveness of the dementia care pathway are diagnoses and time delays (Fig. 1, blue and purple text). Regarding the former, the rates of dementia detection (underdiagnosis) can vary considerably [21], and the diagnosis of dementia, and its subtype, can be inaccurate [22, 23]. In one US study, depending on the permissiveness of clinical and neuropathological criteria, AD diagnosis sensitivity (true positive rate) can range between 71 and 97%, while it is between 44 and 71% for specificity (true negative rate) [24]. Suggested reasons for dementia misdiagnosis include physicians/GPs in primary care not being appropriately trained or confident in detecting the disease (within their brief consultation time) and the lack of standardised validated screening protocols and/or routine implementation of screening [22, 25, 26].

There is also a link between early diagnosis and dementia prevalence. It has been estimated that if early identification of risks and diagnosis, leading to proper treatments or interventions, can delay dementia onset by 2 years, the prevalence would reduce by 20%, with a further prevalence reduction of 50% if a delay of 5 years was achieved [27]. Interestingly, to decrease the national dementia underdiagnosis rate, the UK government has introduced the incentivisation for GPs dementia diagnosis (paid per case); unintended consequences of the approach include poor patient experience, false-positive diagnosis and negative impacts on waiting lists in memory clinics due to increased numbers of referrals [28–30].

Early and accurate diagnosis, on top of providing timely and appropriate care and treatment and reducing undue psychological stress associated with false-positive diagnosis, also has economic benefits. In particular, past studies have shown that patients with prior AD misdiagnosis (false positive) used substantially more medical services until their (non-comorbidity) vascular dementia diagnosis, leading to increased annual medical costs per patient; following corrected diagnosis, the medical costs converged to patients never diagnosed with AD [31, 32].

Regarding the issue of delays in dementia diagnosis, this can be due to various factors. These include false-negative diagnosis, caregivers’ lack of knowledge or reluctance to seek help, uncertainty from patients and families about when and where to seek help, poor communication and uncertainty from medical doctors [22, 33, 34]. For instance, in one review of services in England, waiting times for assessment can range from 3 to 184 days, while dementia diagnosis from referral could take up to 199 days [34]. Such delays could permit substantial cognitive decline. Further, patients identified with MCI have to wait for a follow-up re-evaluation in either a recommended 6-month time interval or when there is a significant change in status [19].

## Assessments in dementia diagnosis

To receive appropriate treatment and support, careful assessment for diagnosing dementia is necessary. Current assessments and their associated ‘markers’ for dementia can comprise several types, from clinical history, biological (e.g. blood- or brain-based) assessment, to neuropsychological and functional assessments (Table 1) [18]. Often, the choice of assessments is based on factors such as accuracy, sensitivity, specificity, cost-effectiveness, and speed and convenience of use.

**Table 1:** Summary of the UK’s primary and secondary (specialist) care diagnosis for people aged 40 years old and over with a suspected diagnosis of dementia [18]

**Primary care diagnosis**	
**Diagnostic variables**	Potential diagnostic variables include the following: • Clinical history • Clinical cognitive assessment • Neuropsychological testing • Physical examination • Medication review
**Secondary (specialist) care diagnosis**	
**Diagnostic variables**	Potential diagnostic variables include the following: • Specified diagnostic criteria • Structural imaging (magnetic resonance imaging (MRI) and computed tomography (CT)) • Single-photon emission computed tomography (SPECT) (e.g. blood flow, dopamine) • Positron emission tomography (PET) (e.g. fluorodeoxyglucose (FDG), amyloid) • Cerebrospinal fluid (CSF) examination • Electroencephalography (EEG) • Brain biopsy • Neuropsychological assessment • Functional assessment • Genetic testing • Neurological examination

Certain assessment types are more costly and less readily available than others. These include cerebrospinal fluid analysis and various neuroimaging modalities in secondary (specialist) care. Moreover, structural neuroimaging is recommended in all cases unless dementia is well advanced and dementia subtype is identified [18]. However, functional neuroimaging is less often conducted to diagnose dementia subtype even though some biomarkers such as beta-amyloid based PET may have the ability to predict the risk of dementia several years prior to the onset of dementia symptoms (albeit with low specificity) [35]. Thus, there is a need to strike a balance among reliable risk prediction, healthcare costs and the inconvenience for the patient. In contrast, blood-based biomarkers have the potential to offer high-throughput data and are easily subjected to repeated measurement even in frail, elderly people. Newer, e.g. neuroinflammatory-based, markers may offer dementia risk prediction at even earlier pre-symptomatic period [14, 36], although the specificity to dementia, and hence practical use, remains unclear.

For cognitive, neuropsychological and functional assessments, some may require the presence of a clinician and nurse, and perhaps caregiver, while others may take a relatively long time to administer; a comprehensive investigation can even go beyond the time frame of a medical appointment [19]. Thus, a balance between convenience and performance of such assessments is required. Interestingly, composite scales, which combine several neurocognitive subscales or with functional activity scales into a single summary score, have recently gathered high interest for preclinical, prodromal and mild AD, especially for early AD therapeutic research [37]. A composite test assesses different domains of cognition and function through the use of discrete subtests and then averages the standard score means from these subsets to yield an overall score [38]. However, it remains unclear whether composites can actually perform better than the current battery of assessments.

In terms of the health economics evidence for these assessments, a number of cost-utility analysis, which report on incremental costs and quality-adjusted life-years (QALYs) analyses, have been conducted [18]. For instance, [39] compared three cognitive and neuropsychological assessments often used by GPs (Mini-Mental State Examination (MMSE), general practitioner assessment of cognition (GPCOG) and 6-item cognitive impairment test (6CIT)) and identified the most cost-effective option (GPCOG), while providing caution regarding the results’ sensitivity to dementia medicines. Similarly, a cost-utility analysis of (beta-amyloid based PET) neuroimaging markers by [40] supported its use in comparison with standard assessment alone or with cerebrospinal fluid (CSF) testing. However, these studies were often limited to a small number of assessments.

Taken together, we have presented several current issues facing dementia assessments and care. In particular, we have emphasised that providing timely and accurate diagnosis is crucial within the dementia care pathway. To improve the effectiveness of dementia diagnosis and care, we shall discuss in the remainder of this review the needs and challenges associated with clinical data transformation and computational approaches in both dementia research and clinical practice. In particular, we shall emphasise the advantages of improving clinical data curation and integration, identifying new dementia markers and assessments through new fundamental sciences and algorithms, and the development of practical decision support systems. These will be discussed along with their challenges.

## Data digitisation, curation and integration

To enable reliable data analyses for evidence-based solutions to improve dementia diagnosis and care, well-curated and “clean” data are necessary. Compliance with some or all of the so-called 5 C’s (clean, consistent, conformed, current and comprehensive) of data quality [41] and appropriate data governance [42] is necessary. Although this is the case in most openly available dementia data acquired within the context of a research study, actual clinical or medical data paints a rather different picture.

A major reason for “dirty” clinical data is due to the lack of standardisation in the dementia care pathway. For instance, in Northern Ireland, although data related to dementia could be formally retrieved and analysed (e.g. through the Health and Social Care Business Services Organisation’s Honest Broker Service), the set of dementia assessments adopted across different practice sites can differ. GPs in England also have similar non-standardisation in dementia assessments [43]. This could be due to the ambiguity within the national (NICE) guidelines, allowing diversity in approaches and locally based “best” practices. When these data are integrated, they can lead to heterogeneity in data variables and systematic missing (“dirty”) data [44–46]. Missing data could also likely arise from other conditions, such as certain individuals being more likely to complete surveys or respond well to questions, individuals late for medical appointments and individuals with severe dementia unable to attend medical appointments altogether. Therefore, practical strategic approaches, e.g. appropriate data cleaning, imputation and harmonisation techniques, are needed before conducting any analysis [47–52]. Indeed, there are some recent and promising large-scale data extraction and integration initiatives such as the UK-CRIS (Clinical Record Interactive Search) system [53] (see below for more examples).

An alternative solution to reduce heterogeneous data is to employ a “small data” approach. As discussed by [54] in this journal’s collection, there are various advantages to this approach, which can uniquely manage complex, dynamic, multi-causal and complex diseases to facilitate individual-level description, prediction and control. Moreover, given the political, institutional and human nature inertia to change, such localisation and decentralisation could actually be a more viable and economical approach, provided the localised data is of sufficient quality. Further, this approach may be suitable to handle known regional variation in the prevalence and detection of dementia associated with the age profile of the population and accessibility to services (see [7, 55] for examples in rural Ireland). Analytical results or models based on such data would also be localised, which may perhaps be more conducive for the practice of personalised or stratified medicine. If data linkages across regional data silos are implemented for analytical insights into wider patterns or trends, similar issues on data integration could arise, as discussed previously.

Clinical or medical data may include unstructured or semi-structured data. For instance, transcription from handwritten notes from clinicians and nurses to consistent digital formats is needed before storing in operational data storage or data mart, and for use in the analysis. With the advent of robust handwriting recognition algorithms, especially deep learning [56], this can be solved to some extent, but medical (e.g. International Classification of Diseases (ICD)) codes may still need to be further decoded in an efficient way. Also, with the increasing use of medical devices such as pervasive (wearable) sensors or detectors that generate continuous data stream and point-of-care technology, real-time signal processing and edge analytics and other big data approaches would be needed [57, 58]. More fundamentally, the way clinical data is captured early on should be changed and formalised to allow better and systematic digitisation of electronic health or medical records. To enable this would require widespread adoption through policy change. Overall, setting a robust and practical data infrastructure is vital for any reliable data analytics or modelling.

## Computational neurology, an integrative computational framework

In [59], we introduced the umbrella term computational neurology to embrace not only computational and theoretical neuroscience, which has largely focused on neural mechanistic or probabilistic modelling [60], but also data-driven artificial intelligence (AI) approaches to handle heterogeneous, complex and large data. Computational or theoretical neuroscience usually requires focused and relatively detailed data (e.g. across neighbouring spatial scales) to model, explain and predict specific biophysics of neural tissues, their activities and functions in either healthy or disordered brains, including in AD and dementia (see e.g. [59, 61–68] and references therein). Such causal-based modelling approaches can help to test hypotheses and elucidate the mechanisms of brain disorders and potential therapeutics.

For such approaches, the required detailed (biological) data may not always be readily available. Further, it may take a long time to realistically model or simulate large-scale brain activities for practical clinical purposes, although there are attempts using simpler reduced computational models [69–71]. Moreover, when data is heterogeneous or when biological information is lacking, biologically realistic mechanistic modelling to bridge across scales may not be feasible, and probabilistic or statistical modelling can be applied. Thus, with the unavailability of mechanistic systems models, causality may be inferred, e.g. based on probabilistic models [60, 72, 73].

When the data gets sufficiently large and complex, the applications of data mining, AI or machine learning become essential. This is especially the case for big data generated by new technologies, as discussed previously. Some of the wider perspectives on this topic have already been discussed in this journal’s collection [74, 75]. Notable open big data initiatives include those for fundamental brain sciences such as the Allen Brain Map [76], Collation of Connectivity Data for the Macaque (CoCoMac) database [77] and Human Connectome Project (HCP) [78], and for clinical and translational sciences, including the Cambridge Centre for Ageing Neuroscience (Cam-CAN) dataset inventory [79], Alzheimer’s Disease Neuroimaging Initiative (ADNI) [80], the National Alzheimer’s Coordinating Center (NACC) [81], UK Biobank [82] and the Dementias Platform UK (DPUK) [83]. Other large-scale projects include those coordinated by the Innovative Medicines Initiative (IMI), e.g. the European Medical Information Framework (EMIF) [84], the European Prevention of Alzheimer’s Dementia Consortium (EPAD) [85], AETIONOMY (Organising mechanistic knowledge about neurodegenerative diseases for the improvement of drug development and therapy) [86] and Neuronet (Efficiently Networking European Neurodegeneration Research) [87].

**Fig. 2:**
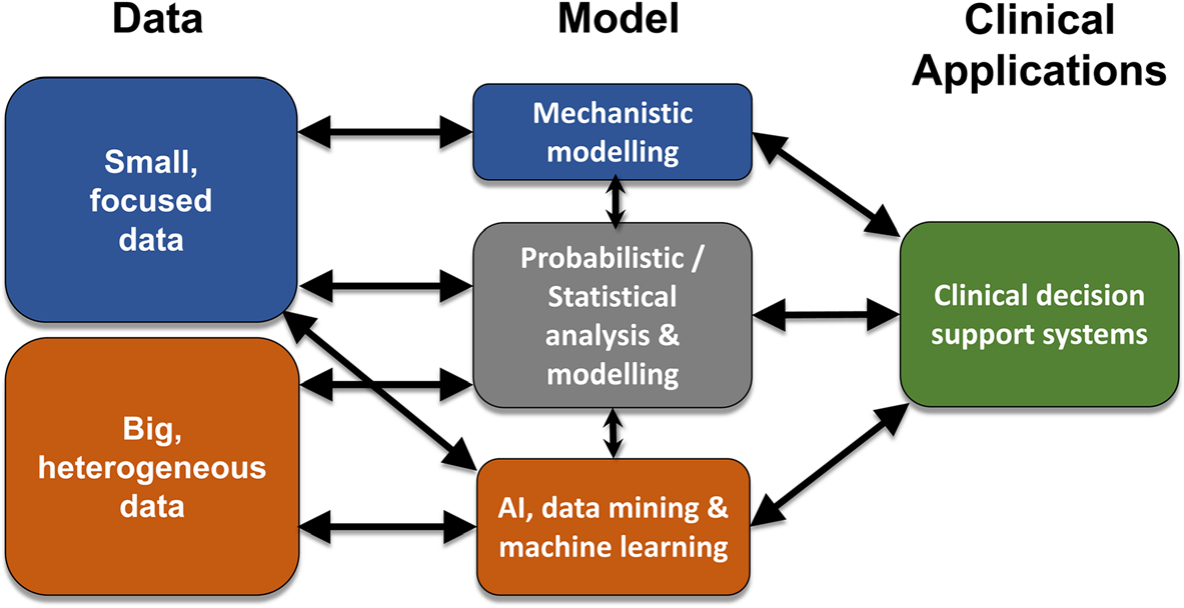
Schematic of computational and theoretical approaches in computational neurology: from fundamental research to clinical applications. Blue boxes: small or focused data; brown boxes: larger or more heterogeneous data. Arrows: relationships. Sometimes, artificial intelligence (AI), data mining and machine learning methods are also used in relatively smaller or less heterogeneous data to guide mechanistic modelling (not shown)

Importantly, these databases and platforms now enable researchers, particularly those with computational or theoretical inclination, to perform large-scale quantitative analyses to enable wider and more direct research impact (e.g. see [88]). There are also opportunities for researchers to link across mechanistic and data-driven computational approaches (e.g. see [89, 90]). Figure 2 summarises the possible interactions of these various modelling approaches with different data types. Together, these computational approaches can be applied for a deeper understanding of dementia, test potential therapeutics and for detecting and predicting dementia.

## Computationally derived and other novel markers of dementia

Computational neurology applied to dementia can potentially solve some of the issues facing dementia diagnosis and prognosis. Particularly, data-driven models can provide more objective methods for detection and risk prediction of dementia. For some applications, the detection accuracy can be higher than that of humans. For instance, in the sub-area of computational neuroimaging, advanced techniques such as deep learning have led to very high accuracy for identifying dementia severity, outperforming human experts [91]. Some neuroimaging work, e.g. [92], has also combined multiple neuroimaging modalities to further enhance dementia predictive accuracy. However, to convince relevant stakeholders of their use in clinical practice, cost-utility analysis of these computational approaches and their identified markers may be needed.

As compared to the current battery of dementia assessments, including recently suggested use of composite scales, computational researchers can now use algorithms to perform unbiased and automated selection of the most relevant assessments or variables, and their (optimal) combinations, for predicting dementia severity and risk (e.g. [73, 88]). Such data-driven approaches may reveal markers that can lie beyond human intuition. Moreover, these computationally derived markers often consist of a smaller number of variables than standard assessments, while still able to provide reasonable (or higher) accurate prediction of dementia. Thus, there is potential that their use can lead to more effective dementia diagnosis.

Novel biomarkers using newer technologies, not currently deployed in the dementia care pathway, may also have the potential to transform dementia diagnosis and prognosis. These include readily accessible novel blood-based markers (using high-throughput next-generation DNA sequencing, proteomic and metabolomic technologies) permitting identification of protein concentrations/activity/isoforms and post-translational modifications, metabolic products, such as amino acids, carbohydrates, lipids, organic acids and nucleic acids (single nucleotide polymorphisms (SNPs)) [93]. Similar data analytical, e.g. feature selection and dimensional reduction, methods can be used to home in and identify key markers [94, 95].

Although not currently part of the dementia care pathway, magnetoencephalography (MEG), with its high temporal resolution, can more directly identify novel biomarkers for dementia and its prodromal stage. They can come in the form of abstract machine learning or functional brain connectivity-based markers [96–99]. Given that electroencephalography (EEG), with poorer spatial localisation than MEG, has already been incorporated in dementia diagnosis (Table 1) [18], it may perhaps be not too inconceivable to also include MEG. Further, MEG, with its ease of use, may be more favourable for frail, elderly or demented participants owing to the avoidance of cumbersome procedures, e.g. preparation of the electrodes and conducting gel as required for EEG. However, the current high costs associated with the acquisition and maintenance of MEG instrumentation impede its widespread use.

Post-clinical validation of computationally derived and other novel markers should be followed by discussion among policymakers, researchers and other stakeholders to allow their assimilation into the current dementia care pathway. For instance, in conjunction with the traditional set of assessments, assessment for novel blood-based markers could be performed using point-of-care technologies within primary care, while MEG assessment conducted at secondary care.

## Practical clinical decision support systems

As of now, and in the foreseeable future, clinicians make an informed clinical diagnosis after weighing over all available diagnostic evidence. Given the complexity of the data forming such evidence and the decision-making processes required, computerised decision support systems (CDSSs) can act as tools to assist human experts with interpretation, diagnosis and treatment [100]. A CDSS may consist of a highly specialised computational model, e.g. for discriminating specific neuroimaging data [101]. It may also consist of systems-based computational model that embraces a wide variety of data types or markers [88, 102]. Crucially, CDSS can act as a bridge from fundamental, data-driven research towards clinical application (Fig. 2).

CDSSs can be useful to solve the underdiagnosis or misdiagnosis of dementia within primary care settings, thereby reducing the load at the secondary care level. In fact, a criticism of the UK’s National Dementia Strategy has suggested that more diagnosis should take place in primary care [34]. Moreover, CDSSs can also provide more effective (e.g. neuroimaging) assessments within secondary care. Further, adoption of a common CDSS platform may promote more standardisation of dementia assessments. When incorporated into the telemedicine scene, the adoption of CDSS could be accelerated through awareness of its resolving of issues in financial costs, delays and accessibility (e.g. in an infectious disease pandemic) related to dementia diagnosis and care. In fact, with the widespread use of smartphones, some dementia assessments may perhaps be digitised and conducted within the CDSS in mobile devices (e.g. the IMI RADAR-AD (Remote Assessment of Disease and Relapse – Alzheimer’s Disease) project [103] and the EDoN (Early Detection of Neurodegenerative diseases) project [104]), increasing accessibility to assessments and expediting early diagnoses in cognitive decline and dementia and other supporting services [105–109]. However, this may also lead to potential data security and privacy issues [58].

While developing computational models for CDSSs, care has to be taken as the models trained in e.g. open dementia datasets may consist of variables (e.g. specific cognitive assessments) that may not be the same as that in clinical practice. Also, individual cases are often not considered in analysis and model validation (but see, e.g. [88]). In longitudinal studies for risk prediction, models need to take into account appropriate time trajectories [110] and trajectory heterogeneity [111]. Thus, many current models’ decisions may have an inappropriate estimation of their predictive precisions for actual clinical practice. Moreover, in open dementia datasets, the proportion of MCI or dementia individuals may not necessarily reflect the actual proportion in society. Thus, an appropriate adjustment may be necessary before translational deployment. In addition, many computational modelling studies often struggle with obtaining high detection accuracy when dealing with MCI cases, regardless of the intrinsic strength of the models (e.g. [91]). This may be due to the studies failing to differentiate the subtypes of MCIs (e.g. amnestic MCI) or the ill-defined general term of MCI [112]. Fundamentally related to this is that the clinical classification of the disease is often mixed. We suggest that the next stage for dementia classification would arise from data-driven computational modelling rather than the standard labels in the Diagnostic and Statistical Manual of Mental Disorders (DSM-5). Particularly, computational neurology could follow the path of computational psychiatry for mental health in the identification of disease categorisation and stages, e.g. through data-driven dimensional or network-based approaches [113, 114].

## Conclusion

Currently, our understanding of dementia is lacking, and the dementia care pathway is suboptimal. We propose that computational neurology approaches can offer specific solutions. With mechanistic biologically based modelling, it can provide insights into the underlying neural mechanisms and assist in dementia therapeutics research. Supported by appropriate data infrastructure, data-driven modelling and CDSS can provide immediate improvements through better dementia diagnosis and prognosis, and improve related care pathways, while potentially reducing delays and health and social care costs. New markers may be elucidated based on algorithms and new technologies, which may complement the current diagnostic and prognostic processes.

However, such benefits may only be realised if computational models and CDSSs are appropriately evaluated and adopted by users. Obstacles to implementation in clinical practice may be explained by the general lack of engagement from clinicians, physicians and health specialists [115]. Indeed, many computational models of dementia may perhaps be too ‘academic’ and lack translational characteristics. To move the field forward, it is imperative that computational researchers, informaticians, clinicians, patients, health institutions, policymakers and other stakeholders should work synergistically together.

## Data Availability

Not applicable.

## References

[1] Gale SA, Acar D, Daffner KR (2018). Dementia. Am J Med.

[2] Dorszewska J, Prendecki M, Oczkowska A, Dezor M, Kozubski W (2016). Molecular basis of familial and sporadic Alzheimer’s disease. Curr Alzheimer’s Res.

[3] Vossel KA, Tartaglia MC, Nygaard HB, Zeman AZ, Miller BL (2017). Epileptic activity in Alzheimer’s diseases: causes and clinical relevance. Lancet Neurol.

[4] Canevelli M, Bruno G, Remiddi F, Vico C, Lacorte E, Vanacore N, Cesari M. Spontaneous reversion of clinical conditions measuring the risk profile of the individual: from frailty to mild cognitive impairment. Front. Med. (Lausanne) 2017;4:184. 10.3389/fmed.2017.00184. 10.3389/fmed.2017.00184 PMC5670103 29164119

[5] World Health Organization. Global Action Plan on the Public Health Response to Dementia, 2017–2025. https:// apps.who.int/iris/bitstream/handle/10665/259615/978924151348 7-eng.pdf Accessed 31 May 2020.

[6] Alzheimer’s Society. Dementia – the true cost: fixing the care crisis. https://www.alzheimers.org.uk/sites/default/files/2018-05/Dementia%20the%20true%20cost%20-%20Alzheimers%20Society%20report.pdf. Accessed 31 May 2020.

[7] Cahill S, O’Shea E, Pierce M. Creating excellence in dementia care: a research review for Ireland’s National Dementia Strategy. 2012. www.icsg.ie, livingwithdementia.tcd.ie. Accessed 31 May 2020.

[8] Pierce M, Cahill S, O’Shea E. Prevalence and projections of dementia in Ireland, 2011-2046, Genio, Mullingar. 2014.

[9] Budson AE, Solomon PR. Cholinesterase inhibitors. Memory Loss, Alzheimer’s Disease, and Dementia, 160–173. Elsevier; 2016.

[10] Cummings J, Passmore P, McGuinness B, Mok V, Chen C, Engelborghs S (2019). Souvenaid in the management of mild cognitive impairment: an expert consensus opinion. Alzheimers Res Ther.

[11] Cummings J, Lee G, Ritter A, Zhong K. Alzheimer’s disease drug development pipeline: 2018. Alzheimer’s Dement. (N.Y.) 2018;4:195–214. 10.1016/j.trci.2018.03.009 PMC6021548 29955663

[12] Makin S (2018). The amyloid hypothesis on trial. Nature.

[13] Mehta D, Jackson R, Paul G, Shi J, Sabbagh M (2017). Why do trials for Alzheimer’s disease drugs keep failing? A discontinued drug perspective for 2010-2015. Expert Opin Investig Drugs.

[14] Du X, Wang X, Geng M (2018). Alzheimer’s disease hypothesis and related therapies. Transl Neurodegener.

[15] Edwards FA (2019). A unifying hypothesis for Alzheimer’s disease: from plaques to neurodegeneration. Trends Neurosci.

[16] World Health Organization, Neurological Disorders: Public Health Challenges. Geneva, World Health Organization, 2006. https://www.who.int/mental_health/neurology/neurodiso/en/. Accessed 31 May 2020.

[17] Alzheimer’s Disease International (2011). World Alzheimer report 2011: the benefits of early diagnosis and intervention.

[18] National Institute for Health and Care Excellence (UK). Dementia: assessment, management and support for people living with dementia and their carers. London: National Institute for Health and Care Excellence (UK); 2018. 30011160

[19] The Dementia Care Pathway. Full implementation guidance. National Collaborating Centre for Mental Health. 2018; https://www.rcpsych.ac.uk/docs/default-source/improving-care/nccmh/dementia/nccmh-dementia-care-pathway-full-implementation-guidance.pdf?sfvrsn=cdef189d_6.

[20] Kane JP, Richardson S, Allan L, Thomas A (2016). Diagnosing dementia. Br J Hosp Med (Lond).

[21] Lang L, Clifford A, Wei L, Zhang D, Leung D, Augustine G (2017). Prevalence and determinants of undetected dementia in the community: a systematic literature review and a meta-analysis. BMJ Open.

[22] Bradford A, Kunik ME, Schulz P, Williams SP, Hardeep S (2009). Missed and delayed diagnosis of dementia in primary care: prevalence and contributing factors. Alzheimer Dis Assoc Disord.

[23] Gaugler JE, Ascher-Svanum H, Roth DL, Fafowora T, Siderowf A, Beach TG (2013). Characteristics of patients misdiagnosed with Alzheimer’s disease and their medication use: an analysis of the NACC-UDS database. BMC Geriatr.

[24] Beach TG, Monsell SE, Phillips LE, Kukull W (2012). Accuracy of clinical diagnosis of Alzheimer’s disease at National Institute on Aging Alzheimer Disease Centers, 2005-2010. J Neuropathol Exp Neurol.

[25] Boise L, Camicioli R, Morgan DL, Rose JH, Congleton L (1999). Diagnosing dementia: perspectives of primary care physicians. Gerontologist.

[26] Moore V, Cahill S (2013). Diagnosis and disclosure of dementia – a comparative qualitative study of Irish and Swedish general practitioners. Aging Ment Health.

[27] Brodaty H, Woolf C, Andersen S, Barzilai N, Brayne C, Cheung KS-L, et al. ICC-dementia (International Centenarian Consortium - dementia): an international consortium to determine the prevalence and incidence of dementia in centenarians across diverse ethnoracial and sociocultural groups. BMC Neurol. 2016;16:52. 10.1186/s12883-016-0569-4. 10.1186/s12883-016-0569-4 PMC4839126 27098177

[28] Larner AJ. Impact of the National Dementia Strategy in a neurology-led memory clinic: 5-year data. Clin. Med. (Lond). 2014;14:216. 10.7861/clinmedicine.14-2-216. 10.7861/clinmedicine.14-2-216 PMC4953305 24715145

[29] Bell S, Harkness K, Dickson JM, Blackburn D (2015). A diagnosis for £55: what is the cost of government initiatives in dementia case finding. Age Ageing.

[30] Liu D, Green E, Kasteridis P, Goddard M, Jacobs R, Wittenberg R (2019). Incentive schemes to increase dementia diagnoses in primary care in England: a retrospective cohort study of unintended consequences. Br J Gen Pract.

[31] Hunter CA, Kirson NY, Desai U, Cummings AK, Faries DE, Birnbaum HG (2015). Medical costs of Alzheimer’s disease misdiagnosis among US Medicare beneficiaries. Alzheimers Dement.

[32] Happich M, Kirson NY, Desai U, King S, Birbaum HG, Reed C (2016). Excess costs associated with possible misdiagnosis of Alzheimer’s disease among patients with vascular dementia in a UK CPRD population. J Alzheimers Dis.

[33] Boise L, Morgan DL, Kaye J, Camicioli R (1999). Delays in the diagnosis of dementia: perspectives of family caregivers. Am J Alzheimers Dis.

[34] Minghella E. Pathways to dementia diagnosis: a review of services in the south-west of England. NHS South of England, Strategic Clinical Network for Mental Health, Dementia and Neurological Conditions South West. 2013. http://dementiapartnerships.com/wp-content/uploads/sites/2/pathwaystodiagnosis.pdf. Accessed 11th June, 2020.

[35] Ewers M, Sperling RA, Klunk WE, Weiner MW, Hampel H (2011). Neuroimaging markers for the prediction and early diagnosis of Alzheimer’s disease dementia. Trends Neurosci.

[36] Quiroz YT, Zetterberg H, Reiman EM, Chen Y, Su Y, Fox-Fuller JT (2020). Plasma neurofilament light chain in the presenilin 1 E280A autosomal dominant Alzheimer’s disease kindred: a cross-sectional and longitudinal cohort study. Lancet Neurol.

[37] Vellas B, Bateman R, Blennow K, Frisoni G, Johnson K, Katz R (2015). Endpoints for pre-dementia AD trials: a report from the EU/US/CTAD Task Force. J Prev Alzheimers Dis.

[38] Schneider LS, Goldberg TE. Composite cognitive and functional measures for early stage Alzheimer’s disease trials. Alzheimers Dement. (Amst). 2020;12:e12017. 10.1002/dad2.12017. 10.1002/dad2.12017 PMC7233425 32432155

[39] Tong T, Thokala P, McMillan B, Ghosh R, Brazier J (2017). Cost effectiveness of using cognitive screening tests for detecting dementia and mild cognitive impairment in primary care. Int J Geriatr Psychiatry.

[40] Hornberger J, Bae J, Watson I, Johnston J, Happich M (2017). Clinical and cost implications of amyloid beta detection with amyloid beta positron emission tomography imaging in early Alzheimer’s disease - the case of florbetapir. Curr Med Res Opin.

[41] Immon WH, Linstedt D (2014). Data architecture: a primer for the data scientist: big data.

[42] Milne R, Brayne C (2020). We need to think about data governance for dementia research in a digital era. Alz Res Therapy.

[43] Koch T, Iliffe S (2011). Implementing the National Dementia Strategy in England: evaluating innovative practices using a case study methodology. Dementia (Lond).

[44] Pedersen AB, Mikkelsen EM, Cronin-Fenton D, Kristensen NR, Pham TM, Pedersen L (2017). Missing data and multiple imputation in clinical epidemiological research. Clin Epidemiol.

[45] Chan K, Fowles JB, Weiner JP (2010). Review: electronic health records and the reliability and validity of quality measures: a review of the literature. Med Care Res Rev.

[46] Kharrazi H, Wang C, Scharfstein D (2014). Prospective EHR-based clinical trials: the challenge of missing data. J Gen Intern Med.

[47] Bath P, Deeg D, Poppelaars J (2010). The harmonisation of longitudinal data: a case study using data from cohort studies in The Netherlands and the United Kingdom. Ageing Soc.

[48] Little RJ, D’Agostino R, Cohen ML, Dickersin K, Emerson SS, Farrar JT (2012). The prevention and treatment of missing data in clinical trials. N Engl J Med.

[49] Miao X, Gao Y, Guo S, Liu W (2018). Incomplete data management: a survey. Front Comput Sci.

[50] Hu Z, Melton GB, Arsoniadis EG, Wang Y, Kwaan MR, Simon GJ (2017). Strategies for handling missing clinical data for automated surgical site infection detection from the electronic health record. J Biomed Inform.

[51] Kourou KD, Pezoulas VC, Georga EI, Exarchos TP, Tsanakas P, Tsinakis M (2019). Cohort harmonization and integrative analysis from a biomedical engineering perspective. IEEE Rev Biomed Eng.

[52] McCombe N, Ding X, Prasad G, Finn DP, Todd S, McClean PL, et al. Predicting feature imputability in the absence of ground truth. In: Proceedings of the 37^th^ International Conference on Machine Learning (ICML): the Art of Learning with Missing Values (ARTEMISS) Workshop, Vienna, Austria, 17 July, 2020.

[53] Clinical Record Initiative Search system (CRIS). https://crisnetwork.co/. Accessed 31 October 2020.

[54] Hekler EB, Klasnja P, Chevance G, Golaszewski NM, Lewis D, Sim I (2019). Why we need a small data paradigm. BMC Med.

[55] Alzheimer’s Society (2014): Dementia 2014: Opportunity for change. https://www.alzheimers.org.uk/sites/default/files/migrate/downloads/dementia_2014_opportunity_for_change.pdf.

[56] LeCun Y, Bengio Y, Hinton G (2015). Deep learning. Nature.

[57] Chen L, Hoey J, Nugent CD, Cook DJ, Yu Z. Sensor-based activity recognition. IEEE Trans. Syst. Man Cybern., Part C (Applications and Reviews). 2012;42:790–808.

[58] Ienca M, Vayena E, Blasimme A. Big data and dementia: charting the route ahead for research, ethics, and policy. Front Med (Lausanne). 2018;5:13. 10.3389/fmed.2018.00013. 10.3389/fmed.2018.00013 PMC5808247 29468161

[59] Wong-Lin K, Sanchez-Bornot JM, McCombe N, Kaur D, McClean PL, Zou X, et al. Computational neurology: computational modeling approaches in dementia. In: Wolkenhauer, Olaf (ed.). Systems medicine: integrative, qualitative and computational approaches, vol. 2, pp. 81-89. Oxford: Elsevier.

[60] Dayan P, Abbott LF. Theoretical neuroscience. MIT Press. 2001.

[61] Zou X, Coyle D, Wong-Lin K, Maguire L (2011). Computational study of hippocampal-septal theta rhythm changes due to ß-amyloid-altered ionic channels. PLoS One.

[62] Zou X, Coyle D, Wong-Lin K, Maguire L (2012). Beta-amyloid induced changes in A-type K+ current can alter hippocampo-septal network dynamics. J Comput Neurosci.

[63] Abuhassan K, Coyle D, Belatreche A, Maguire L (2014). Compensating for synaptic loss in Alzheimer’s disease. J Comput Neurosci.

[64] Cutsuridis V, Moustafa AA (2016). Multiscale models of pharmacological, immunological and neurostimulation treatments in Alzheimer’s disease. Drug Discov Today Dis Model.

[65] Cutsuridis V, Moustafa, A. Computational models of Alzheimer’s disease Scholarpedia 2017;12: 32144. 10.4249/scholarpedia.32144.

[66] Cutsuridis V, Moustafa, AA. Computational models of pharmacological and immunological treatment in Alzheimer’s disease, in: Computational models of brain and behavior. John Wiley & Sons, Ltd, Chichester, UK 2017:99–108. 10.1002/9781119159193.ch8.

[67] Hassan M, Abbas Q, Seo SY, Shahzadi S, Al Ashwal H, Zaki N (2018). Computational modeling and biomarker studies of pharmacological treatment of Alzheimer’s disease (review). Mol Med Rep.

[68] Joshi A, Wang DH, Watterson S, McClean PL, Behera CK, Sharp T (2020). Opportunities for multiscale computational modelling of serotonergic drug effects in Alzheimer’s disease. Neuropharmacology..

[69] Rystar R, Fornari E, Frackowiak RS, Ghika JA, Knyazeva MG (2011). Inhibition in early Alzheimer’s disease: an fMRI-based study of effective connectivity. Neuroimage.

[70] Penny W, Iglesias-Fuster J, Quiroz YT, Lopera FJ, Bobes MA (2018). Dynamic causal modeling of preclinical autosomal-dominant Alzheimer’s disease. J Alzheimers Dis.

[71] Alderson TH, Bokde ALW, Kelso JAS, Maguire L, Coyle D (2018). Metastable neural dynamics in Alzheimer’s disease are disrupted by lesions to the structural connectome. Neuroimage.

[72] Pearl J. Causality: models, reasoning and interference. 2^nd^ Ed., Cambridge University Press. 2009.

[73] Ding X, Bucholc M, Wang H, Glass DH, Wang H, Glass DH (2018). A hybrid computational approach for efficient Alzheimer’s disease classification based on heterogeneous data. Sci Rep.

[74] Kelly CJ, Karthikesalingam A, Suleyman M, Corrado G, King D (2019). Key challenges for delivering clinical impact with artificial intelligence. BMC Med.

[75] Car J, Sheikh A, Wicks P, Williams MS (2019). Beyond the hype of big data and artificial intelligence: building foundations for knowledge and wisdom. BMC Med.

[76] Allen Brain Map. https://portal.brain-map.org/. Accessed 31 Oct 2020.

[77] Collation of Connectivity Data for the Macaque (CoCoMac) database. http://cocomac.g-node.org/main/index.php. Accessed 31 Oct 2020.

[78] Human Connectome Project (HCP). http://www.humanconnectomeproject.org/. Accessed 31 Oct 2020.

[79] Cambridge Centre for Ageing Neuroscience (Cam-CAN) dataset inventory https://www.cam-can.org/. Accessed 31 Oct 2020.

[80] Alzheimer’s Disease Neuroimaging Initiative (ADNI). http://adni.loni.usc.edu/. Accessed 31 Oct 2020.

[81] National Alzheimer’s Coordinating Center (NACC). https://www.alz.washington.edu/. Accessed 31 Oct 2020.

[82] UK Biobank. https://www.ukbiobank.ac.uk/. Accessed 31 Oct 2020.

[83] Dementias Platform UK (DPUK). https://www.dementiasplatform.uk/. Accessed 31 Oct 2020.

[84] European Medical Information Framework (EMIF). https://www.imi.europa.eu/projects-results/project-factsheets/emif. Accessed 31 Oct 2020.

[85] European Prevention of Alzheimer’s Dementia Consortium (EPAD). http://ep-ad.org/. Accessed 31 Oct 2020.

[86] AETIONOMY (Organising mechanistic knowledge about neurodegenerative diseases for the improvement of drug development and therapy). https://www.imi.europa.eu/projects-results/project-factsheets/aetionomy. Accessed 31 Oct 2020.

[87] Neuronet (Efficiently Networking European Neurodegeneration Research). https://www.imi-neuronet.org/. Accessed 31 Oct 2020.

[88] Bucholc M, Ding X, Wang H, Glass DH, Wang H, Prasad G (2019). A practical computerized decision support system for predicting the severity of Alzheimer’s disease of an individual. Expert Syst Appl.

[89] Geerts H, Dacks PA, Devanarayan V, Haas M, Khachaturian ZS, Gordon MF (2016). Big data to smart data in Alzheimer’s disease: the brain health modeling initiative to foster actionable knowledge. Alzheimers Dement.

[90] Alber M, Tepole AB, Cannon WR, De S, Dura-Bernal S, Garikipati K (2019). Integrating machine learning and multiscale modeling - perspectives, challenges, and opportunities in the biological, biomedical, and behavioral sciences. NPJ Digit Med.

[91] Qiu S, Joshi PS, Miller MI, Xue C, Zhou X, Karjadi C, et al. Development and validation of an interpretable deep learning framework for Alzheimer’s disease classification. Brain. 2020;awaa137. 10.1093/brain/awaa137. 10.1093/brain/awaa137 PMC7296847 32357201

[92] Youssofzadeh V, McGuinness B, Maguire LP, Wong-Lin K (2017). Multi-kernel learning with Dartel improves combined MRI-PET classification of Alzheimer’s disease in AIBL data: group and individual analyses. Front Hum Neurosci.

[93] Hampel H, O’Bryant SE, Molinuevo JL, Zetterberg H, Masters CL, Lista S (2018). Blood-based biomarkers for Alzheimer disease: mapping the road to the clinic. Nat Rev Neurol.

[94] Saeys Y, Inza I, Larrañaga P (2007). A review of feature selection techniques in bioinformatics. Bioinformatics.

[95] Leclercq M, Vittrant B, Martin-Magniette ML, Scott Boyer MP, Perin O, Bergeron A (2019). Large-scale automatic feature selection for biomarker discovery in high-dimensional OMICs data. Front Genet.

[96] Koelewijn L, Lancaster TM, Linden D, Dima DC, Routley BC, Magazzini L (2019). Oscillatory hyperactivity and hyperconnectivity in young *APOE*-ɛ4 carriers and hypoconnectivity in Alzheimer’s disease. Elife..

[97] Yang S, Bornot JMS, Wong-Lin K, Prasad G (2019). M/EEG-based bio-markers to predict the MCI and Alzheimer’s disease: a review from the ML perspective. IEEE Trans Biomed Eng.

[98] Maestú F, Fernández A (2020). Role of magnetoencephalography in the early stages of Alzheimer disease. Neuroimaging Clin N Am.

[99] Sanchez-Bornot JM, Lopez ME, Bruna R, Maestú F, Youssofzadeh V, Yang S, Finn DP, Todd S, McLean PL, Prasad G, Wong-Lin K. High-dimensional brain-wide functional connectivity mapping in magnetoencephalography. J Neurosci Methods. 2020;108991. 10.1016/j.jneumeth.2020.108991. 10.1016/j.jneumeth.2020.108991 33181166

[100] Sim I, Gorman P, Greenes RA, Haynes RB, Kaplan B, Lehmann H (2001). Clinical decision support systems for the practice of evidence-based medicine. J Am Med Inform Assoc.

[101] Hu C, Ju R, Shen Y, Zhou P, Li Q. Clinical decision support for Alzheimer’s disease based on deep learning and brain network. In: Proceedings of the IEEE International Conference on Communications (ICC), Kuala Lumpur, 2016, 2016:1–6, 10.1109/ICC.2016.7510831.

[102] Bruun M, Frederiksen KS, Rhodius-Meester HFM, Baroni M, Gjerum, L, Koikkalainen J, et al*.* Impact of a clinical decision support tool on prediction of progression in early-stage dementia: a prospective validation study. Alzheimers Res. Ther. 2019;11:25 (2019). 10.1186/s13195-019-0482-3. 10.1186/s13195-019-0482-3 PMC6425602 30894218

[103] IMI RADAR-AD (Remote Assessment of Disease and Relapse – Alzheimer’s Disease) project. https://www.radar-ad.org/. Accessed 31 Oct 2020.

[104] EDoN (Early Detection of Neurodegenerative diseases) project. https://edon-initiative.org/. Accessed 31 Oct 2020.

[105] Newman C, Hodges J, Pearson S, Noad R. The design and implementation of a computer supported assessment of dementia-ACEmobile. Int J Integr Care. 2014;14. 10.5334/ijic.1784.

[106] Gholipour B (2016). Can you diagnose dementia from a gaming app? Scientific American.

[107] Carvalho CM, Christina D, Saade M, Conci A, Seixas FL, Laks J. A clinical decision support system for aiding diagnosis of Alzheimer’s disease and related disorders in mobile devices. In: Proceedings of the 2017 IEEE International Conference on Communications (ICC), Paris, 2017; 2017:1–6, 10.1109/ICC.2017.7996968.

[108] Muniz-Terrera G, Watermeyer T, Danso S, Ritchie C (2019). Mobile cognitive testing: opportunities for aging and neurodegeneration research in low- and middle-income countries. J Glob Health.

[109] Kourtis LC, Regele OB, Wright JM, Jones GB (2019). Digital biomarkers for Alzheimer’s disease: the mobile/wearable devices opportunity. NPJ Digit Med.

[110] Kaur D, Bucholc M, Finn DP, Todd S, Wong-Lin K, McClean PL. Multi-time-point data preparation robustly reveals MCI and dementia risk factors. Alzheimers dementia (Amst);12(1):e12116. 2020. 10.1002/dad2.12116. 10.1002/dad2.12116 PMC7560502 33088897

[111] René JFM, Haaksma ML, Nuiz-Terrera G (2019). Understanding and predicting the longitudinal course of dementia. Curr Opin Psychiatry.

[112] Saunders S, Ritchie K, Russ T, Muniz-Terrera G (2018). Ritchie C (2018). Evolution and future directions for the concept of mild cognitive impairment. Int. Psychogeriatr..

[113] Huys QJ, Maia TV, Frank MJ (2016). Computational psychiatry as a bridge from neuroscience to clinical applications. Nat Neurosci.

[114] Heinz A. An understanding of mental disorders: computational models for dimensional psychiatry. The MIT Press. 2017.

[115] Khairat S, Marc D, Crosby W, Sanousi AAS (2018). Reasons for physicians not adopting clinical decision support systems: critical analysis. JMIR Med Inform.

